# St. Thomas's Hospital and the Victory Loan

**Published:** 1917-02-24

**Authors:** 


### St. Thomas's Hospital and the Victory Loan.

A successful effort has been made at St. Thomas's Hospital during the past week to help the War Loan, and the staff who contributed have to be congratulated very warmly on their effort to give every assistance in their power. It is said sometimes by soldiers from the Front and others, whose efforts to help their country in this great crisis of their national history cannot be commended too highly, "We have done our bit; it is not for us to do any more." This certainly is not the view held at St. Thomas's. At no hospital in the country has the staff "done its bit" more successfully and with greater self-negation. How readily the Governors came forward in the first few days after the declaration of war and gave 200 beds to the service of the country free of all cost during the earlier months of the war, how willingly the senior staff have gone on service, how large a number of the qualified nursing staff volunteered for immediate service abroad, and how generously those who were left met the emergency and filled the openings is well known to all. This did not suffice, and during the country's great effort of last week the Governors, with a view to helping all who desired to do something more, expressed to the staff their willingness to advance money free of all interest for investment in the War Loan, to be repaid out of salaries or wages during the year.

£3,067 for the War.

Unfortunately, owing to the date of the meeting, this decision of the Governors could not be made known until Wednesday, February 14. The nurses were then called together by the matron, and the decision of the Governors was communicated to them by the secretary. These officials were helped by the matron's assistant, with the result that the nurses put down their names for £1,430 Registered Stock. The office staff and workmen of the hospital took another £520 of Registered Stock, and the nurses took a further 178 War Savings Certificates, for all of which the Governors advanced a total sum of £1,990. In addition to those purchased with the money advanced by the Governors, nurses took out through their War Savings Association 258 certificates. Thus a total contribution of £2,386 was subscribed to the War Loan by or on behalf of the employees of St. Thomas's Hospital in two days. Including the £681 already invested through their War Savings Association, the grand total is reached of £3,067 invested in War Loans, an achievement of which all the staff of St. Thomas's may well be proud.

